# Effect of *Anacardium occidentale* Leaf Powder on Growth Performance, Diarrhea Incidence, Blood Biochemistry, and Intestinal Traits in Weaned Piglets

**DOI:** 10.3390/ani14233382

**Published:** 2024-11-25

**Authors:** Roisbel Aroche, Ge Gao, Yanpin Li, Yonggang Zhang, Román Rodríguez, Yordan Martínez, Xilong Li

**Affiliations:** 1 Key Laboratory of Feed Biotechnology of the Ministry of Agriculture and Rural Affairs, Institute of Feed Research, Chinese Academy of Agricultural Sciences, Beijing 100081, China; rarocheg@gmail.com (R.A.); 82101192355@caas.cn (G.G.); liyanpin@caas.cn (Y.L.); 2 Department of Animal Husbandry, Faculty of Agricultural Sciences, University of Granma, Bayamo 85100, Cuba; 3 Huanshan Group Co., Ltd., Qingdao 266000, China; zhangyg22@vanke.com; 4 Animal Production Study Center, Faculty of Agricultural Sciences, University of Granma, Bayamo 85100, Cuba; rrodriguezb@udg.co.cu; 5 Faculty of Veterinary Medicine, University of Fondwa, Léogâne 6210, Haiti

**Keywords:** *Anacardium occidentale* leaf, diarrhea, gut health, piglets

## Abstract

To evaluate the phytobiotic effect of *Anacardium occidentale* leaf powder (AOLP) on growth performance, diarrhea incidence, blood biochemistry, and intestinal traits, seventy-two weaned piglets were randomly distributed into four groups (six replicates/group and three pigs/replicate) for 28 days, receiving a control diet (T0) or being supplemented with 5 (T1), 10 (T2), or 15 (T3) g/kg of AOLP. The diets did not affect the growth performance (*p* > 0.05); however, the AOLP groups had a decreased diarrhea incidence and malondialdehyde concentration (*p* < 0.05; 28 days). However, the AOLP groups had increased immunoglobulins (G and M) and villus heights (*p* < 0.05) in the duodenum. Likewise, T3 improved the number of goblet cells in the villi and the whole intestine (*p* < 0.01), the Mucin2 area in the jejunum and ileum (*p* < 0.05), occludin gene expression in the jejunum (*p* < 0.01), and acetic and valeric acid production (*p* < 0.05). Microbial diversity at the genus level was not different (*p >* 0.05); however, T3 increased the abundance of the *Lactobacillus* genus. These findings suggest that dietary supplementation with AOLP improved intestinal health by increasing antioxidant, immune, anti-inflammatory, and antidiarrheal activity in the weaned piglets.

## Simple Summary

The weaning period is a critical phase in pig production due to many factors predisposing piglets to gastrointestinal disorders, mainly diarrheal syndrome. This study focused on the use of *Anacardium occidentale* (*A. occidentale*) leaf powder (AOLP) as a phytobiotic supplement for weaned piglets. The results showed that the natural product (*A. occidentale*) did not modify the productivity of the piglets; however, it reduced the diarrhea rate, perhaps due to the increase in intestinal histomorphometry, the gene expression of binding proteins, the goblet cell number, intestinal Mucin2, cecal acetic acid, and plasma immunoglobulins (G and M) and the decrease in oxidative stress. Also, although microbial diversity did not change due to the effect of the experimental diets, a higher relative abundance of the *Lactobacillus* genus was found. These findings suggest that the use of a natural product obtained from *A. occidentale* leaf powder may be beneficial for the health of piglets, due to its immunological, antioxidant, anti-inflammatory, antimicrobial, and antidiarrheal effects, potentially leading to more sustainable practices in pig production and animal welfare.

## 1. Introduction

The weaning period is a critical phase in pig production, marked by important physiological and environmental changes such as feed transition, separation from the mother, and regrouping with other piglets, which predisposes them to suffering gastrointestinal disorders, such as diarrheal syndrome [1,2]. Diarrhea is the loss of electrolytes and water in liquid and semi-liquid feces, which, although not considered a disease, can cause dehydration, induce diseases, and compromise animal welfare, affecting the feed efficiency of piglets and increasing their mortality and production costs [3,4].

Although many investigations have recommended using various nutritional strategies to control diarrheal syndrome in piglets, such as diets rich in dietary fiber and lower in crude protein, it remains a challenge for the industry and scientists [5,6]. Currently, the industry uses antibiotic growth promoters in water or feed in permitted countries or recommends the use of natural alternatives with antioxidant, anti-inflammatory, and antimicrobial activities [7,8]. These natural alternatives such as prebiotics, probiotics, postbiotics, synbiotics, organic acids, and phytobiotics aim to mitigate the negative impact of weaning stress on piglet health and performance, thereby promoting productivity and profitability in the swine industry [2,9,10].

In this context, one of the most prominent phytobiotic products that have been investigated is the *Anacardium occidentale* tree. It is a perennial plant that adapts to many regions, and several of its in vitro properties such as the antioxidant and antimicrobial properties of the leaf powder have been described, since it can counteract oxidative stress and inhibit the growth of pathogenic microorganisms [11,12,13]. Furthermore, in vivo studies have shown that the dietary supplementation of AOLP alone or mixed with other medicinal plants had a positive impact on the growth and health status of broiler chickens, laying hens, and weaned piglets [14,15,16]. In this sense, dietary supplementation with AOLP in poultry promoted viability, productivity, edible portions, and immune function and increased the relative weight of viscera and immune organs [14,16]. The dietary inclusion of this natural product decreased the incidence of diarrhea in lactating pigs and improved the efficiency of pigs in the growing–fattening phase [17].

However, the process underlying the improvement of productive indicators in pigs by AOLP remains poorly understood. Thus, the present study aims to evaluate the effect of dietary supplementation with AOLP on the growth performance, diarrhea incidence, blood biochemistry, and intestinal traits of weaned piglets. In this way, it is possible to explore its potential as an enhancer of pig productivity and health, particularly during the challenging transition period of weaning.

## 2. Materials and Methods

The animal protocol for this research was approved by the Animal Care and Use Committee of the Institute of Feed Research of the Chinese Academy of Agricultural Sciences (IFR-CAAS20230926).

### 2.1. Experimental Design and Animal Management

The experiment was carried out at the Experimental Station in Langfang from the Feed Research Institute of the Chinese Academy of Agricultural Sciences. A total of 72 healthy weaned piglets ([Yorkshire × Landrace] × Duroc) with similar initial body weights (BW, 8.03 ± 0.31 kg) and ages (28 ± 1 days) were randomly assigned to four diets. The dietary treatments consisted of a control (T0) and supplementation with 5 (T1), 10 (T2), or 15 (T3) g/kg of AOLP, respectively.

Each treatment had 6 replicate pens with 3 piglets per replicate. During the experiment, the piglets had ad libitum access to feed and water. A combination of daylight and artificial light with 12 h of illumination was used, and ventilation was provided by variable-speed fans. The temperature was adjusted weekly from 28 °C at the start to 26 °C at the end of the experiment. The pens (1.4 × 1.7 m^2^) had a slatted floor design, and each pen was equipped with a feeder and two water nipples. The diets were formulated according to the National Research Council’s [18] nutrient requirements (Table 1) and were manufactured one week before starting the trial without the inclusion of any antibiotic growth promoter. The diets’ levels of crude protein, calcium, and phosphorus were determined as described by Jiang et al. [19].

### 2.2. Growth Performance and Diarrhea Incidence Measurements

Body weight was recorded at day 0 (weaning day), day 14, and day 28 (final day). Feed intake was recorded for each pen every 2 wks. Growth performance was assessed by measuring the average daily gain (ADG), average daily feed intake (ADFI), and gain-to-feed intake ratio (G–F) for each pen. Diarrhea incidence was determined by monitoring the fecal scores of each animal daily using a five-point fecal consistency scoring system: 1 = hard (dry pellet); 2 = firm (formed stool); 3 = soft (moist stool that retains its shape); 4 = soft (unformed stool); and 5 = unformed watery liquid. A fecal score of 4–5 was considered indicative of diarrhea [19,20]. The diarrhea incidence (%) was calculated as [(number of weaned piglets with diarrhea × number of days of diarrhea)/(total number of weaned piglets × number of days of the experiment)] × 100. After recording and statistically analyzing the productive performance and diarrhea incidence in the four experimental treatments, group T3 was chosen for subsequent animal sampling and comparative analysis.

After knowing the results of the growth performance and diarrheal syndrome in this study and considering other in vitro and in vivo studies from our laboratory with this natural product (AOLP) or others in monogastric animals, groups T0 and T3 were selected for the following laboratory analyses [15,21].

### 2.3. Sample Collection

Blood was collected on the mornings of days 14 and 28, via venipuncture from the jugular vein, from a randomly selected piglet per pen. Blood samples in heparin tubes were centrifuged at 3000× *g* for 10 min at 4 °C to obtain plasma and immediately stored at −20 °C for further analysis. Additionally, on day 28, fecal samples were collected from each piglet and were immediately carefully bagged, labeled, and placed at −20 °C to stop fermentation. On day 28, one piglet per pen was stunned using a portable electrical stunner (with 220 V as the output power). To collect the tissues, the abdomen was longitudinally incised, and sections of the small intestine were preserved in fresh 4% paraformaldehyde for subsequent analyses. Samples of the jejunum and ileum mucosa were then placed in cryogenic vials (Corning Incorporated, New York, NY, USA), quickly frozen in liquid nitrogen, and stored at −80 °C until future analysis in the lab.

### 2.4. Antioxidant Indexes in Plasma

Antioxidant indexes were measured in the plasma by spectrophotometry using commercial assay kits (Nanjing Jiancheng Bioengineering Institute, Nanjing, China) according to the manufacturer’s instructions. The activity of catalase (CAT) was assessed using ammonium molybdate, monitoring the changes in absorbance at a wavelength of 405 nm. For glutathione peroxidase (GSH-Px), 5,50-dithiobis-p-nitrobenzoic acid was utilized and noted the absorbance shift at 412 nm. To evaluate the superoxide dismutase (SOD) activity, a nonenzymatic nitroblue tetrazolium (NBT) test was used, which detects the inhibition of superoxide anion free radicals that interact with nitroblue tetrazolium in a sample. The change in absorbance at 450 nm was recorded. The total antioxidant capacity (T-AOC) was determined through the 2,2′-azino-bis (3-ethylbenzothiazoline-6-sulfonic acid) (ABTS) method, and the absorbance was measured at 405 nm. The concentration of malondialdehyde (MDA) was measured by 2-thiobarbituric acid, and the absorbance shift was recorded at 532 nm.

### 2.5. Immunoglobulin Measurement in Plasma

The plasma concentration of IgA, IgG, and IgM were determined using commercial ELISA kits (Nanjing Jiancheng Bioengineering Institute, Nanjing, China) according to the manufacturer’s instructions. Standard curves were created by performing serial dilutions of the recombinant porcine immunoglobulin. The results were reported in mg/mL, referencing the established standard curve for accuracy.

### 2.6. Intestinal Morphology

Samples of the duodenum, jejunum, and ileum were collected from six animals per treatment, were dehydrated using a series of increasing concentrations of ethanol, were then clarified with xylene, and were then embedded in paraffin. Subsequently, three sections of a 5 μm thickness were cut from the intestinal samples and stained with hematoxylin-eosin to assess the intestinal morphology. For the goblet cell counts, the paraffin sections from the jejunum and ileum were treated with a staining technique that combines alcian blue and periodic acid–Schiff (AB-PAS), and anti-MUC2 rabbit pAB was applied (Servicebio Technology Co., Ltd., Wuhan, China). The slides were observed under an optical microscope for the villus height (VH) and crypt depth (CD) (500 µm at 4×) and for the goblet cell count (20 µm at 100×) (Olympus CX43, Olympus Corporation, Tokyo, Japan). Ten areas were randomly chosen for measurements of the VH and CD and to count the goblet cells from the villus and the crypts using an Image Pro-Plus 6.0 Software Analysis System (Media Cybernetics, Singapore). The villus height–crypt depth ratio (V–C) and the total goblet cell count were calculated. For the purpose of statistical analysis, the average of these values was used.

### 2.7. Mucin2 Area Determination

Paraffin was removed from sections by immersing them in xylene and was rehydrated through a decreasing ethanol series (100, 95, 80 and 70%) and finally rinsed in distilled water. For Mucin2 staining, heat-induced antigen retrieval was performed to enhance the Mucin2 accessibility. Endogenous peroxidase activity and nonspecific binding sites were blocked and sections were incubated with a specific primary antibody. A secondary antibody conjugated to horseradish peroxidase was applied. Diaminobenzidine (DAB) was used as the chromogen, producing a brown signal at the sites of Mucin2. Sections were counterstained with hematoxylin to visualize cell nuclei and were dehydrated and mounted. Stained sections were imaged using an optical microscope at a 10× magnification (Olympus CX43, Olympus Corporation, Tokyo, Japan) and the images were imported into ImageJ v1.8.0 software (NIH, Bethesda, MD, USA). The color deconvolution plugin in ImageJ was used to separate the DAB (Mucin2) and hematoxylin components. A threshold was applied to segment the DAB-stained area, and the ImageJ measurement tools were utilized to quantify the area corresponding to Mucin2.

### 2.8. RNA Extraction and Quantitative Real-Time PCR (qPCR)

The extraction of total RNA from the jejunum and ileum mucosa was performed with TRIzol (Thermo Fisher Scientific, Inc., Boston, MA, USA) following the manufacturer’s instructions. For tissue homogenization, 50 mg of the samples was added to 1 mL of lysis buffer, and 5 min of incubation at room temperature followed. Then, 0.2 mL of chloroform was added, and the samples were shacked for 15 s and incubated for 3 min at room temperature. Later, the samples were centrifuged at 12,000 rpm (Hitachi, Koki Co., Ltd., Tokyo, Japan) at 4 °C for 10 min. Afterwards, the supernatant was collected and an equal volume of anhydrous ethanol was added, and the samples were mixed well, transferred to an RA absortion column, and centrifuged at 12,000 rpm for 45 s. Next, 0.5 mL of RE deproteinization solution was added, the samples were centrifuged at 12,000 rpm for 45 s, and the waste liquid was discarded. Then, 0.5 mL of RW wash solution was added and the samples were centrifuged at 12,000 rpm for 45 s, and this step was repeated once. Finally, the samples were centrifuged at 13,000 rpm for 2 min, had 60 μL of Rnase-free water added, were let to stand at room temperature for 2 min, and were centrifuged at 12,000 rpm for 1 min, and the sample RNA was obtained. The concentration and quality of the RNA were analyzed by an Epoch microplate spectrophotometer, and the A260/A280 ratio was determined for additional determinations. Then, total RNA (1 µg) was used for cDNA reverse transcription using a TransScript First-Strand cDNA Synthesis Kit (TransGen Biotech, Beijing, China). SYBR Green reagent was used for the RT-qPCR (Thermo Fisher Scientific, Inc., Boston, MA, USA) using a CFX96 Touch real-time PCR instrument (Bio-Rad Laboratories Inc., Berkeley, CA, USA). The target genes’ relative expression was calculated using the 2^−∆∆CT^ method, and the housekeeping gene was glyceraldehyde-3-phosphate dehydrogenase (GAPDH). The sequences of used primers are shown in Table 2.

### 2.9. Analysis of Microbiota Diversity

Microbial diversity within the fecal samples was assessed by employing Majorbio’s established methodology. This study focused on the V3 and V4 regions of the 16S rDNA gene, using the universal primers 338F (ACTCCTACGGGAGGCAGCAG) and 806R (GGACTACHVGGGTWTCTAAT) to amplify these specific segments. Subsequently, the sequencing of the PCR products was conducted on the Illumina MiSeq PE300 platform (San Diego, CA, USA). Raw sequence data underwent quality filtration utilizing Fast software (version 0.19.6). Subsequent to high-quality sequence de-noising, amplicon sequence variants (ASVs) were identified. To maintain uniformity, the number of reads per sample was standardized to 4000, resulting in an average output of 97.90%. The analysis of the ASV data was carried out using the Majorbio cloud platform (Bio-Pharm, Technology Co., Shanghai, China), adhering to the standard procedures outlined by Majorbio Biopharm Technology Co., Ltd. (Shanghai, China).

### 2.10. Short-Chain Fatty Acids (SCFA) Concentration

The fecal samples were dissolved in a precooled dilution of water (including ZnSO_4_·7H_2_O and K_4_Fe (CN)_6_·3H_2_O), centrifuged (at 4 °C for 10 min at 10,000 rpm), and filtered; then, the supernatant was diluted using distilled water at a 1:4 radio. At this stage, the supernatant was analyzed for SCFA using an 883 Ion Chromatograph (IC; Metrohm, Herisau, Switzerland).

### 2.11. Statistical Analysis

Data from the growth performance was processed with ANOVA using the GLM procedure. To evaluate the effects of different levels of AOLP supplementation, orthogonal polynomial comparison tests with linear and quadratic effects were used, where the pen represented the experimental unit. Diarrhea incidence was anallyzed by the chi-square test. The plasma antioxidant indexes, plasma immunoglobulin, intestinal morphology, goblet cell count, Mucin2 area determination, gene expression, and SCFA production were analyzed by an independent-samples *t*-test, where individual piglets represented the experimental unit. Differences were considered statistically significant at *p* ≤ 0.05. The software used for all the analyses was the SPSS (IBM® SPSS® Statistics, version 27.0 (2020), SPSS Inc., Chicago, IL, USA).

## 3. Results

### 3.1. Growth Performance and Diarrhea Incidence

The effects of dietary supplementation with AOLP on the growth performance of the weaned piglets are shown in Table 3. The dietary treatments did not significantly change (*p* > 0.05) the productive indicators (BW, ADG, ADFI, and G–F ratio) of the weaned pigs. However, a quadratic effect was found (*p* < 0.05) on the G–F during days 0 to 14. There does not appear to be a linear relationship between increasing the levels of this natural product (*A. occidentale*) and feed efficiency.

Table 4 indicates that dietary supplementation with AOLP decreased (*p* < 0.05) the diarrhea incidence by 10.14 (54.90 %), 9.12 (49.38 %), and 12.87 (69.68 %) percentual units for days 14–28 and 5.63 (44.58 %), 5.13 (40.62 %), and 4.47 (35.39 %) for days 0–28 for T1, T2, and T3 compared to T0, respectively. It is noteworthy that the treatments with this medicinal powder supplementation (*A. occidentale*) showed no difference between themselves for this indicator (*p* > 0.05).

### 3.2. Antioxidant Status

**Figure 1:**
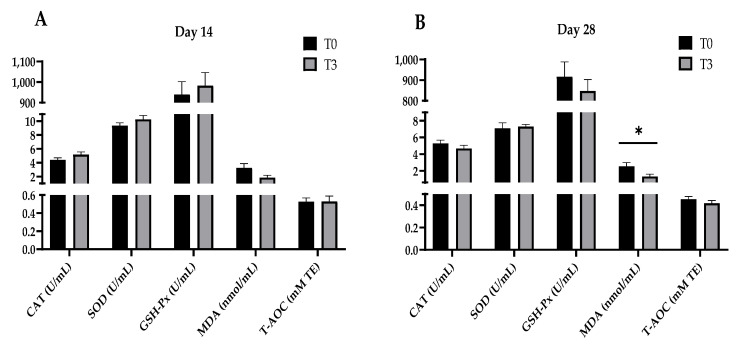
Effect of dietary supplementation with AOLP on plasma antioxidant indexes of weaned piglets on day 14 (**A**) and day 28 (**B**). Results are presented as mean ± SEM; n = 6. * *p* < 0.05 indicates difference between treatments. T0 = control group; T3 = group supplemented with 15 g/kg AOLP; CAT = catalase; SOD = superoxide dismutase; GSH-Px = glutathione peroxidase; MDA = malondialdehyde; and T-AOC = total antioxidant capacity.

Figure 1 shows that increasing the levels of AOLP supplementation did not modify the plasma antioxidant indexes (CAT, SOD, GSH-Px, MDA, and T-AOC) of the weaned pigs after 14 days of starting the test (*p* > 0.05). However, after 28 days, the T3 group’s plasma MDA concentration decreased (*p* < 0.05) compared to the control group (*p* < 0.05), with no changes (*p* > 0.05) between the treatments for the other plasma antioxidant indicators (CAT, SOD, GSH-Px, and T-AOC).

### 3.3. Immunoglobulin Concentration in Plasma

**Figure 2:**
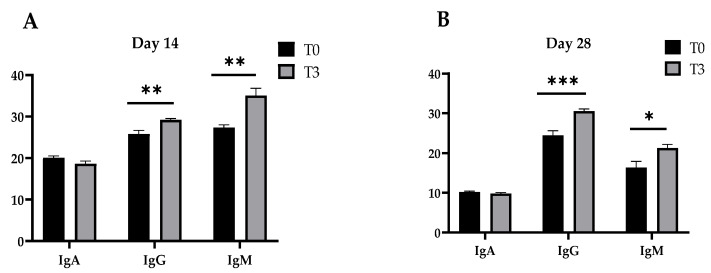
Effect of dietary supplementation with AOLP on plasma immune concentration (mg/mL) of weaned piglets on day 14 (**A**) and day 28 (**B**). The results are presented as mean ± SEM; n = 6. * *p* < 0.05; ** *p* < 0.01; and *** *p* < 0.001 indicate difference between treatments. T0 = control group; T3 = group supplemented with 15 g/kg AOLP.

The concentrations of the immunoglobulins (A, G, and M) of the pigs fed a control diet and with a dietary supplementation of 15 g/kg of AOLP are shown in Figure 2. After 14 and 28 days, T3 provoked an increase in the plasma concentration of IgG and IgM (*p* < 0.05) in the weaned pigs compared to the control diet, although IgA remained unchanged between treatments (*p* > 0.05) in the periods measured (14 and 28 days).

### 3.4. Intestinal Morphology and Goblet Cell Number

**Figure 3:**
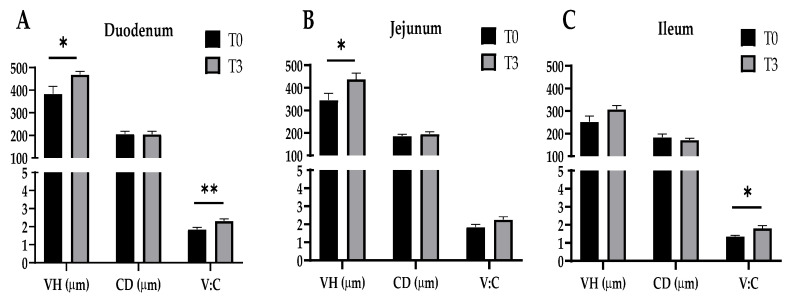
Effects of dietary supplementation with AOLP on morphology of duodenum (**A**), jejunum (**B**), and ileum (**C**) of weaned piglets. VH, villus height; CD, crypt depth; V–C, villus height-to-crypt depth ratio. Results are presented as mean ± SEM; n = 6. * *p* < 0.05 and ** *p* < 0.01 indicate difference between treatments. T0 = control group; T3 = group supplemented with 15 g/kg AOLP.

**Figure A1:**
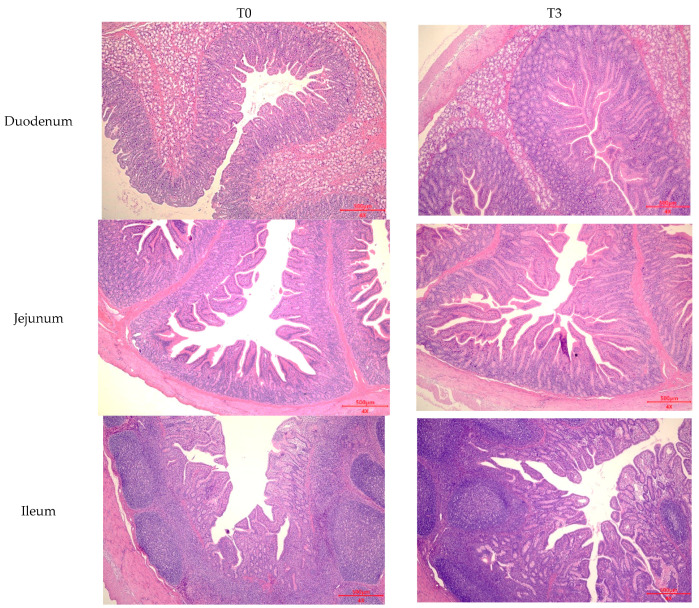
Effects of dietary supplementation with AOLP on intestinal morphology of weaned piglets.

The effect of dietary supplementation with 15 g/kg of AOLP on the small intestinal morphology of the weaned piglets is shown in Figure 3 and Figure A1. In the duodenum, the AOLP supplementation significantly improved the VH and V–C (*p* < 0.05) compared to the control. Furthermore, in the jejunum and ileum, T3 alone modified the VH and V–C compared to the diet without additives, respectively (*p* < 0.05). In the intestinal portions, the CD was not significantly modified (*p* > 0.05) due to the experimental treatments.

**Figure 4:**
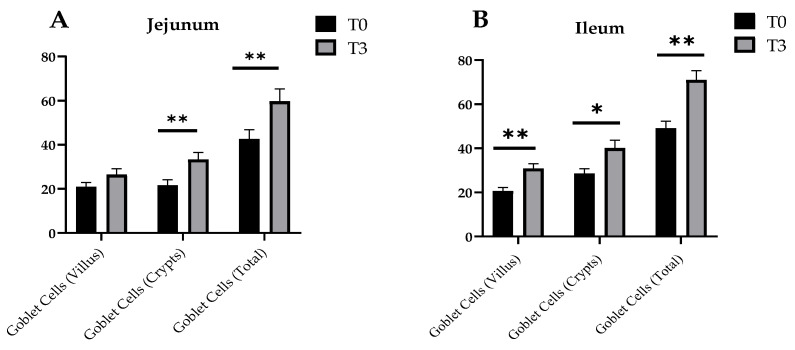
Effects of dietary supplementation with AOLP on number of goblet cells of jejunum (**A**) and ileum (**B**) of weaned piglets. Results are presented as mean ± SEM; n = 6. ** *p* < 0.01 and * *p* < 0.05 indicate difference between treatments. T0 = control group; T3 = group supplemented with 15 g/kg AOLP.

**Figure A2:**
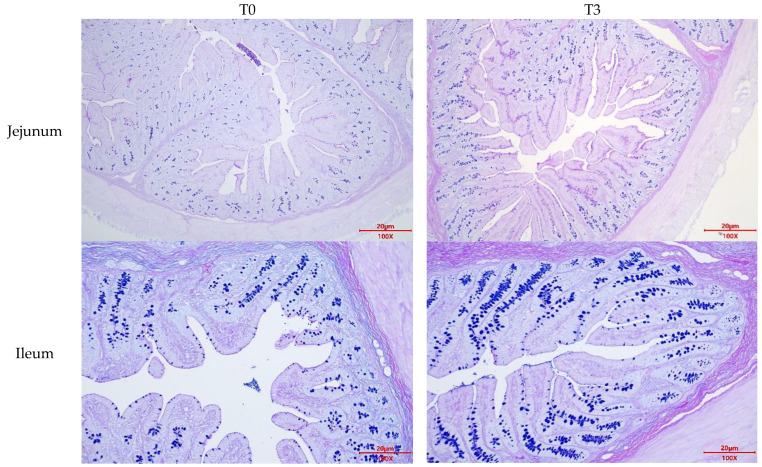
Effects of dietary supplementation with AOLP on goblet cell number of weaned piglets.

Changes in the goblet cell number of the small intestine of the weaned piglets due to dietary supplementation with AOLP is shown in Figure 4 and Figure A2. In the jejunum, T3 significantly stimulated (*p* < 0.05) the goblet cells in the crypts and the total goblet cells, although the goblet cells in the villus were not different between treatments (*p* > 0.05). In the ileum, all the measured indicators were significant between treatments (*p* < 0.05), with higher values for the group with the supplementation of AOLP.

**Figure 5:**
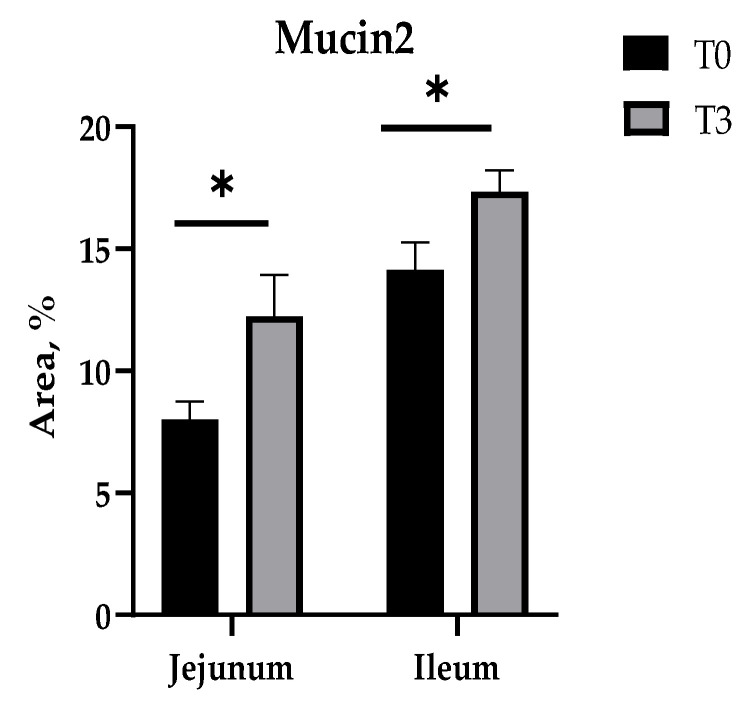
Effects of dietary supplementation with AOLP on intestinal Mucin2 area. Results are presented as mean ± SEM; n = 6. * *p* < 0.05 indicates difference between treatments. T0 = control group; T3 = group supplemented with 15 g/kg AOLP.

**Figure A3:**
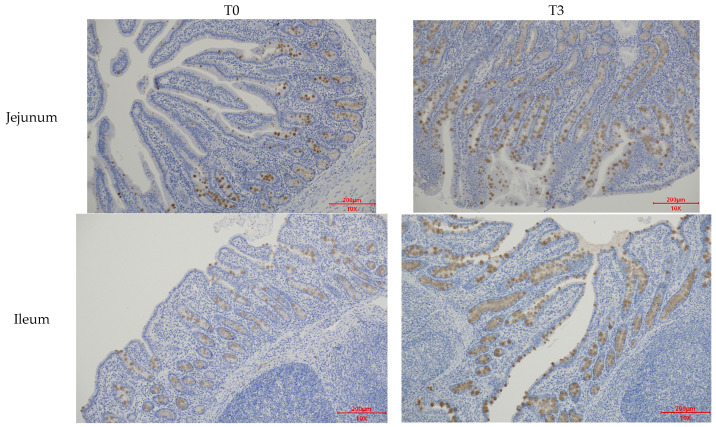
Effects of dietary supplementation with AOLP on intestinal Mucin2 area of weaned piglets.

Furthermore, in these intestinal sections (the jejunum and ileum), it is observed that T3 increased the percentage of Mucin2 to a higher value in relation to the control (Figure 5 and Figure A3).

### 3.5. Relative Gene Expression

**Figure 6:**
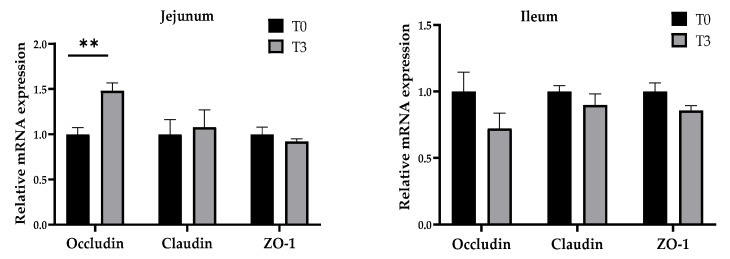
Effects of dietary supplementation with AOLP on mucosal relative gene expression in jejunum and ileum. Results are presented as mean ± SEM; n = 6. ** *p* < 0.01 indicates difference between treatments. T0 = control group; T3 = group supplemented with 15 g/kg AOLP.

As shown in Figure 6, dietary supplementation with 15 g/kg of AOLP significantly increased (*p* < 0.05) the gene expression of occludin in the jejunum mucosa, whereas no changes (*p* > 0.05) were seen in the gene expression of claudin and ZO-1. The ileum mucosa gene expression showed no significant difference for the occludin, claudin, and ZO-1 between both groups (*p* > 0.05).

### 3.6. Microbiota Diversity

**Figure 7:**
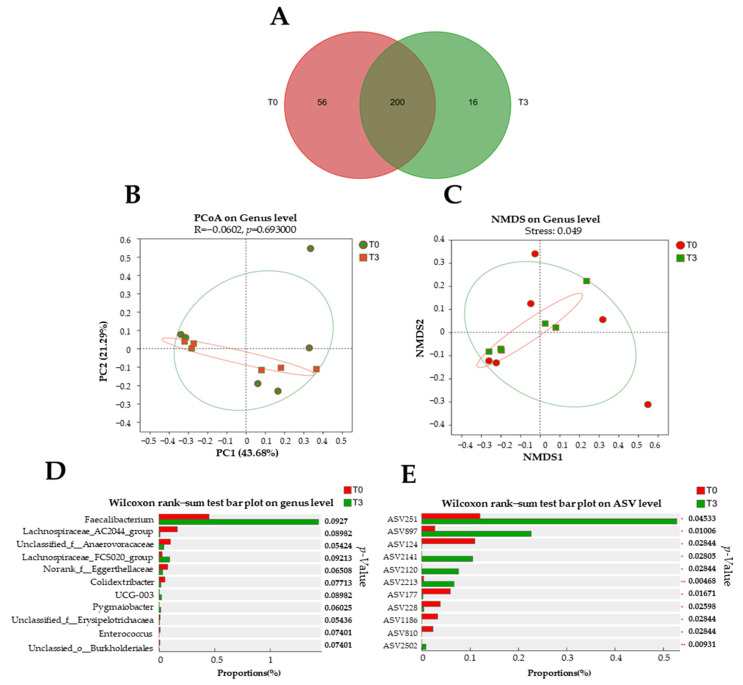
Effect of dietary supplementation with AOLP on fecal microbiota of weaned piglets (n = 6). (**A**) Venn diagram of genera in fecal samples. (**B**) Non-metric multidimensional scaling on genus level. (**C**) Principal coordinate analysis. (**D**) Wilcoxon rank–sum test bar plot on genus level and (**E**) amplicon sequence variants (ASV) level. T0 = control group; T3 = group supplemented with 15 g/kg AOLP.

Around 200 bacterial genera were found between the T0 and T3 groups (Figure 7A). However, there were 56 unique bacterial genera in the T0 group and 16 unique bacterial genera in the T3 group. No difference was found (*p* > 0.05) in the microbiota population at the genus level with the dietary supplementation with 15 g/kg of AOLP compared to the control group (Figure 7B,C).

Although a tendency (*p* < 0.1) is shown in the community barplot analysis (Figure 7D), significant differences are shown on the Wilcoxon rank–sum test bar plot on ASV (Figure 7E). In the group with the dietary supplementation with AOLP, ASVs such as 251, 897, 2141, 2120, 2213, and 2502 were decreased (*p* < 0.05), whereas ASVs 124, 177, 228, 810, and 1186 were increased (*p* < 0.05).

**Figure 8:**
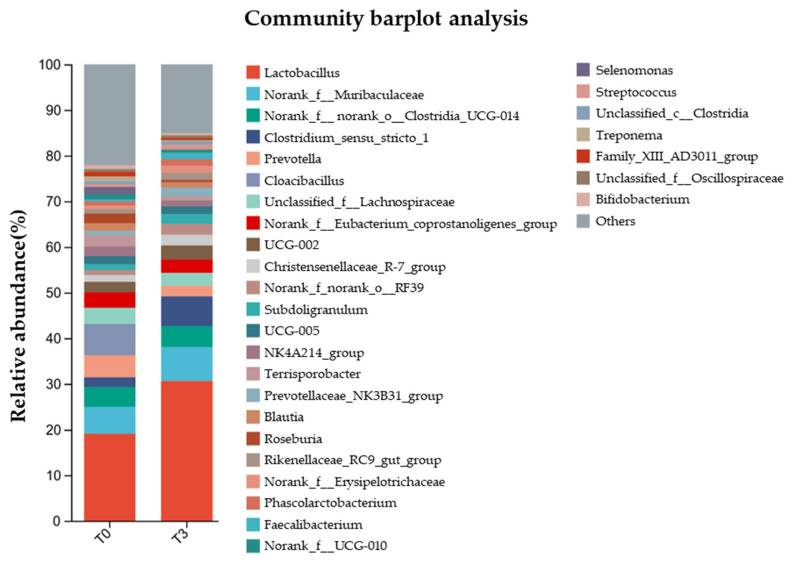
Effect of dietary supplementation with AOLP on relative abundance of gut microbiota at genus level.

Meanwhile, the relative abundance of gut microbiota at the genus level (Figure 8) showed that *Lactobacillus* were the main bacteria in the communities, where it represented less than 20% in the T0 group and more than 30% in the T3 group.

### 3.7. SCFA Production

**Figure 9:**
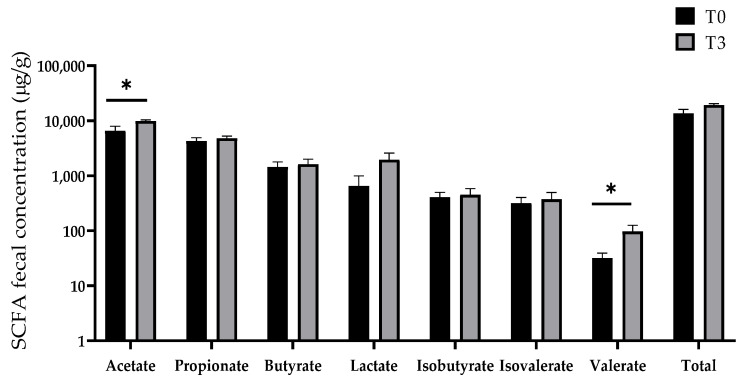
Effect of dietary supplementation with AOLP on SCFA production of weaned piglets. Results are presented as mean ± SEM; n = 6. * *p* < 0.05 indicates difference between treatments. SCFA = short-chain fatty acid.

Figure 9 displays the effect of dietary supplementation with 15 g/kg of AOLP on the SCFA concentration of the weaned piglets. The concentration of acetate and valerate in the T3 group was significantly higher (*p* < 0.05) than in the control group. No changes were seen in the production of propionate, butyrate, lactate, isobutyrate, and isovalerate (*p* > 0.05).

## 4. Discussion

The main objective of the experiment was to study the biochemical, physiological, microbiological, and immunological mechanisms involved in the growth of weaned pigs fed with *A. occidentale* supplements. Previous studies have shown that the dietary inclusion of *A. occidentale*, alone or mixed with other medicinal plants, does not cause morbidity and mortality in pigs; on the contrary, this medicinal powder (AOLP) promotes productive performance and decreases the diarrhea incidence in pre-weaned and post-weaned piglets [17]. Likewise, Aroche et al. [15] found that dietary supplementation with 10 and 15 g/kg of mixed leaf powder from *A. occidentale*, *Psidium guajava*, *Moringa oleifera*, and *Morinda citrifolia* (40:20:20:20%, respectively) promoted growth performance and controlled diarrheal syndrome in weaned pigs. In this study, dietary supplementation with 5, 10, and 15 g/kg of *A. occidentale* did not significantly modify the supplemented pigs’ growth performance compared to the control group; this may be due to the difference in the supplementation levels, environment, number of animals used, and duration of the trial.

Diarrhea poses a significant risk to piglets during the weaning phase. This stage is marked by various physiological and environmental stress factors that are crucial for the growth and overall health of pigs, ultimately impacting the swine industry [22]. In the present study, the medicinal powder obtained from the leaves of *A. occidentale* decreased the diarrhea incidence in these animals, particularly in the last two weeks, indicating that this medicinal powder has chemical compounds with antimicrobial, anti-inflammatory, and astringent properties [23,24,25]. In previous research conducted by our team, we reported a high quantification of several chemical compounds present in AOLP, mainly quercetin, quercetin 3-O-glucoside-7-O-rhamnoside, chicoric acid, kaempeferol-7-O-glucoside, caffeic acid, and cinnamic acid. Also, this natural product decreased the in vitro growth of strain of *E. coli*, *S. aureus*, *S. enteritidis*, and *S. Typhimurium*, as well as having a high antioxidant activity similar to butylated hydroxytoluene (BHT) [21]. Similarly, Duangjan et al. [24], Sunderam et al. [23], and Siracusa et al. [25] reported that the leaves of *A*. *occidentale* have phenolic compounds such as gallic acid, catechin, tannic acid, rutin, quercetin, isoquercetin, chicoric acid, hydroquinine, kaempferol, caffeic acid, and cinnamic acid, among others, which contribute to the medicinal effect attributed to this plant [26,27]. The results from this study indicated that *A*. *occidentale* may be an antidiarrheal additive for weaning piglets.

Intestinal morphology plays a vital role in nutrient absorption, but weaning stress leads to notable alterations in intestinal structure and function in mammals, including villus atrophy, crypt hyperplasia, reduced digestibility, and a compromised intestinal barrier integrity and immune function [28,29]. In this sense, the AOLP positively modified intestinal morphometry, with emphasis on the VH and V–C ratio, in the small intestines of the weaned piglets, suggesting that the chemical compound from the leaves of this plant have beneficial effects for intestinal structure and function. Many studies have recommended the use of subtherapeutic antibiotics or natural alternatives such as probiotics, postbiotics, organic acids, prebiotics, and phytobiotics to alleviate these adverse effects [2,30]. In this regard, several studies have shown that medicinal plants used as extracts or integrals and rich in secondary metabolites positively change the intestinal integrity of weaned pigs [31,32,33]. The research by Sun et al. [34] found that the addition of 0.5, 1.0, and 2.0% of Xiasangju residues (classic traditional Chinese medicine formula composed of *Prunellae spica*, *Morus alba* leaves, and *Chrysanthemi indici* Flos, rich in compounds such as rutin, luteolin, and quercetin) to the diet of weaned piglets decreased the diarrhea score, while supplementation with 0.5, 1.0, 2.0, and 4.0% of Xiasangju residues increased the VH and V–C and decreased the CD in the jejunum and ileum. Ma et al. [29] found that the dietary inclusion of rutin (phenolic compound) decreased the diarrhea index and increased the G–F, jejunal villus height, and V–C. In another study, Deng et al. [32] tested the effect of tannic acid derived from gallnut supplementation on the growth performance and health status of weaned piglets and found that they could increase the VH and V–C and decrease the CD in the small intestine. These results demonstrate that dietary supplementation with 15 g/kg of AOLP improves the intestinal health of pigs in the post-feeding stage, since it positively modifies intestinal histomorphometry, perhaps due to the decrease in intestinal inflammation, which increases the absorption of nutrients and reduces the incidence of diarrheal syndrome in these animals.

Also, maintaining a balanced antioxidant system is crucial for stable intestinal health in weaned piglets. Reactive oxygen species contribute to maintain high inflammation levels in the intestine, resulting in a higher diarrhea incidence [35]. In our study, the dietary supplementation with 15 g/kg of AOLP decreased the MDA concentration in the plasma on day 28. This result is closely related to the antioxidant effect of this natural product (AOLP), rich in phenolic compounds and flavonoids, as it can mitigate lipid peroxidation and oxidative stress [36]. Furthermore, it seems that its dietary use for 28 days exacerbated the antioxidant effect of this natural product, which could mitigate MDA production during the weaning period (the most critical period). According to Deng et al. [32] dietary supplementation with 3 g/kg of tannic acid decreased MDA concentration in the jejunum of weaned piglets. Likewise, Li et al. [37] found that *Perilla frutescens* seed extract, which is rich in polyphenols and flavonoids, decreased MDA in the liver of weaned piglets when included in their diet. Similarly, Xiao et al. [38] found that the dietary inclusion of 0.1% ellagic acid decreased serum MDA and increased SOD. Furthermore, Zhu et al. [33] demonstrated that the inclusion of licorice extract in the diets of weaned piglets decreased serum MDA and GSH-Px. The authors justified their findings by pointing to the wide variety of polyphenolic compounds, flavonoids, and other antioxidant components of this natural product. Decreased MDA production from using 15 g/kg of AOLP influenced diarrheal syndrome outcomes and intestinal integrity, confirming that AOLP has a direct effect on intestinal health in weaned pigs.

Immune status is essential for intestinal balance in weaned piglets. It is known that IgM is the first antibody produced in response to an initial infection. It is effective in forming antigen–antibody complexes and activating the complement system [39]. However, IgG is the most abundant antibody in serum and plays a crucial role in the secondary immune response [40]. It provides long-term immunity and memory after infection or vaccination [41]. In the present study, the concentration of IgG and IgM increased with the dietary supplementation of 15 g/kg of AOLP during the experimental period; these results influenced the lower incidence of diarrhea in the T3 group. Other studies with medicinal plants have also demonstrated the efficacy of including natural compounds in the diet of farm animals to improve their growth performance and health status [34,42,43]. Sun et al. [34] found that supplementation with different levels of Xiasangju residues increased the serum IgG, IgM, and IgA levels of weaned piglets, although no changes were found in ileum sIgA levels. In this regard, Zeng et al. [42] reported that serum IgA and IgG increased in weaned piglets when a dietary inclusion of 8 g/kg of pomelo (*Citrus paradisi*) peel powder was provided. Another study aimed to evaluate the effects of hawthorn and yam (*Dioscorea opposite* Thunb.) extract compounds (rich in polyphenols and flavonoids) on the immune status of weaned piglets, and the results showed an increase in serum IgA and IgG levels compared to the control group [43]. These findings suggest that phytobiotic compounds rich in secondary metabolites used in low concentrations can improve the immune system of weaned piglets, which benefits animal welfare [44]. This shows that *A. occidentale* also has a marked immunological effect, mainly modulating humoral immunity, which gives it greater protection against pathogenic microorganisms, especially in weaned pigs, which are more prone to gastrointestinal disorders.

Additionally, this study assessed the number of goblet cells to evaluate the functional response of the intestinal epithelium to AOLP supplementation. Goblet cells are specialized for the synthesis and secretion of mucus, which serves to inhibit bacterial penetration. This mucus is continuously renewed, facilitating the expulsion of bacteria toward the lumen. In this sense, T3 also improved the number of goblet cells and increased the area of Mucin2 in the jejunum and ileum. These results confirm the direct effect of this natural product (AOLP) on intestinal health, since goblet cells and the mucus produced are an essential part of innate immune defenses and are strongly related to the adaptive immune system [45]. Likewise, the mucus of goblet cells is abundant in Mucin2, which is one of the most abundant mucins present in the intestinal lumen of pigs [46]; therefore, a more robust intestinal morphology structure causes better nutrient absorption and less diarrhea in piglets, as occurred in the T3 group. Furthermore, these results may be related to gut-associated lymphoid tissue (GALT), which is an essential component of the immune system that generates both humoral and cellular responses [41].

In this sense, the intestinal barrier is tightly controlled by junction proteins, such as occludin, which is a transmembrane protein [1]. In this experiment, T3 stimulated occludin gene expression in the jejunum, suggesting that *A. occidentale* strengthened intestinal barriers and intestinal permeability due to the stimulation of local immune responses by immune cells, such as T cells and macrophages, and decreased pathogen translocation [47]. These results confirm that dietary supplementation with 15 g/kg of AOLP increases intestinal protection by different physiological pathways in weaned pigs, both by increasing intestinal permeability and by the higher preservation of epithelial barrier function [29,48,49]. All these findings explain the lower incidence of diarrhea in the T3 group compared to the control group.

Furthermore, maintaining a stable microbial diversity is crucial for gut health, allowing the prevention of the overgrowth of pathogenic bacteria and maintaining the overall functionality of the gut ecosystem [50,51]. The fact that this medicinal powder (AOLP) improved specific markers of gut health suggests that dietary supplementation with this natural product, rich in beneficial secondary metabolites with a marked antidiarrheal effect, did not cause detrimental changes in the gut microbiome of the weaned pigs. These beneficial bacteria play a key role in digestion, immune modulation, and protection against pathogens [51].

Another important role of the intestine is to concentrate the waste from the digestion process in the large intestine [52]. The cecum and colon accumulate more than 500 species of bacteria to degrade feed residues coming from the small intestine. The proportion of each species depends on the characteristics of the diet. However, during weaning, a bacterial imbalance is very common, leading to a higher incidence of diarrhea and less time for feed digestion in the intestine [2]. In our experiment, dietary supplementation with AOLP not only decreased diarrhea (Table 4) but also increased the concentration of acetate and valerate, with a slightly higher concentration of lactate and total SCFA. These effects indicate that the fermentation of residual feed was better in the experimental group than in the control group, which was also the group with the highest diarrhea incidence. Acetate is the main SCFA produced in the large intestine, followed by propionate and butyrate [5]. Higher SCFA production with this pattern (acetate/propionate/butyrate) facilitates a lower pH, which inhibits the overgrowth of pathogenic bacteria [5,53]. However, the higher valerate concentration in the T3 group indicated that more fiber entered the cecum and colon of this group [5], due to the nature of the AOLP. Thus, microbial diversity was not different for both treatments (T0 vs. T3); however, T3 caused a higher population of *Lactobacillus*, which is a genus of lactic acid bacteria (LAB) that produce short-chain fatty acids and decrease intestinal pH to cause competitive exclusion. Thus, AOLP can stimulate the proliferation of this beneficial bacteria directly or indirectly. This study indicates that the supplementation of 15 g/kg AOLP had a strong phytobiotic activity because it improved the conditions for the growth of *Lactobacillus* in the colon, modulating the immune and antioxidant responses and reducing intestinal disorders and the incidence of diarrhea in the weaned piglets. However, further research is needed to elucidate the effect of different AOLP supplementation in pigs challenged with lipopolysaccharides (LPS) or pathogenic bacteria, as well as the use of this natural product in other pig categories and its effect on carcass yield and meat quality. It would also be interesting to obtain and purify the main secondary metabolites in different extracts (alcoholic, aqueous, and ethereal) of this medicinal plant (*A*. *occidentale*) in order to know the effect of these products on the productivity and health of pigs under different production conditions.

## 5. Conclusions

Dietary supplementation with AOLP in weaned piglets decreased the incidence of diarrhea, without changing the productivity of the pigs. Dietary supplementation with 15 g/kg of AOLP) decreased the plasma concentration of MDA and increased the concentrations of IgG and IgM, as well as improving the intestinal integrity and the production of acetate and valerate in the large intestines of the pigs.

## Figures and Tables

**Table 1:** Ingredients and nutrient composition of basal diets (as-fed basis).

Ingredients (%)	Pre-Starter (d 0–14)	Starter (d 14–28)
Corn	48.04	61.32
Soybean meal	13.50	18.00
Extruded soybean	12.00	5.00
Fish meal	5.60	3.00
Whey	15.00	5.00
Soybean oil	1.30	1.30
Monocalcium phosphate	0.35	0.60
Limestone (CaCO_3_)	0.77	0.95
Salt	0.40	0.40
L-lysine HCL	1.10	1.03
DL-methionine	0.08	0.07
Threonine	0.26	0.24
Tryptophane	0.02	0.01
Wheat bran	0.11	1.81
Choline chloride (60%)	0.05	0.05
Phytase	0.02	0.02
Acidifier	0.20	0.20
Premix ^1^	1.00	1.00
Zinc oxide	0.20	-
Total	100.00	100.00
Analyzed nutrient levels, %		
Crude protein	20.26	18.53
Calcium	1.12	0.82
Phosphorus	0.75	0.58
Calculated nutrient content		
ME ^2^, kcal/kg	3350	3310
SID lysine ^3^, %	1.30	1.15
SID methionine, %	0.38	0.34
SID threonine, %	0.76	0.68
SID tryptophan, %	0.21	0.19
SID valine, %	0.82	0.73
SID isoleucine, %	0.69	0.59

^1^ Premix supplied per kilogram of diet: vitamin A, 35.2 mg; vitamin D_3_, 7.68 mg; vitamin E, 128 mg; vitamin K_3_, 8.16 mg; vitamin B_1_, 4 mg; vitamin B_2_, 12 mg; vitamin B_6_, 8.32 mg; vitamin B_12_, 4.8 mg; niacin, 38.4 mg; calcium pantothenate, 25 mg; folic acid, 1.68 mg; biotin, 0.16 mg; iron (FeSO_4_·H_2_O), 171 mg; zinc (ZnSO_4_·H_2_O), 110 mg; manganese (MnSO_4_·H_2_O), 42.31 mg; copper (CuSO_4_·5H_2_O), 125 mg; selenium (Na_2_SeO_3_), 0.19 mg; cobalt (CoCl_2_), 0.19 mg; iodine (Ca(IO_3_)_2_), 0.54 mg. ^2^ ME = metabolizable energy. ^3^ SID = standardized ileal digestibility.

**Table 2:** List of primer sequences used for quantitative real-time PCR.

Gene	Accession Number	Primer Sequence (5′-3′)	Size (bp)
*GAPDH*	NM_001206359.1	F: GCTTGTCATCAATGGAAAGG	86
		R: CATACGTAGCACCAGCATCA	
*Occludin*	NM_001163647.2	F: TCAGGTGCACCCTCCAGATT	112
		R: TGGACTTTCAAGAGGCCTGG	
*Claudin-1*	NM_001244539.1	F: CCTCAATACAGGAGGGAAGC	76
		R: CTCTCCCCACATTCGAGATGATT	
*ZO-1*	CV870309	F: CGATCACTCCAGCATACAAT	111
		R: CACTTGGCAGAAGATTGTGA	

**Table 3:** Effect of dietary supplementation with AOLP on growth performance of weaned piglets.

	Experimental Treatments							
Items	T0	T1	T2	T3	SEM	*p*-Value		
						ANOVA	Linear	Quadratic
Body Weight (kg)								
Day 0	8.03	8.03	8.03	8.03	0.31	1.000	0.953	0.988
Day 14	10.72	10.68	10.50	10.59	0.32	0.952	0.666	0.835
Day 28	14.19	14.32	14.16	15.46	0.52	0.458	0.230	0.384
Average Daily Gain (g)								
Days 0–14	192	189	177	183	9.59	0.951	0.655	0.838
Days 14–28	248	260	261	348	24.54	0.478	0.195	0.461
Days 0–28	220	225	219	265	11.41	0.441	0.222	0.372
Average Daily Feed Intake (g)								
Days 0–14	267	299	261	251	10.38	0.484	0.417	0.354
Days 14–28	449	495	474	598	28.39	0.147	0.054	0.404
Days 0–28	358	397	368	425	13.99	0.233	0.132	0.720
Gain–Feed Intake Ratio								
Days 0–14	0.72	0.63	0.65	0.73	0.02	0.096	0.722	0.017
Days 14–28	0.46	0.51	0.55	0.58	0.03	0.717	0.266	0.869
Days 0–28	0.60	0.57	0.60	0.63	0.02	0.793	0.511	0.497

T0: control; T1: 5 g/kg of AOLP; T2: 10 g/kg of AOLP; T3: 15 g/kg of AOLP. SEM = standard error of the mean.

**Table 4:** Effect of dietary supplementation with AOLP on diarrhea incidence of weaned piglets (%).

	Experimental Treatments				
Items	T0	T1	T2	T3	*p*-Value
Days 0–14	6.35	5.16	5.15	9.92	0.106
Days 14–28	18.47 ^a^	8.33 ^b^	9.35 ^b^	5.60 ^b^	<0.001
Days 0–28	12.63 ^a^	7.00 ^b^	7.50 ^b^	8.16 ^b^	0.007

^a,b^: mean values with different superscript letters within a row were significantly different (*p* < 0.05). T0: control; T1: 5 g/kg of AOLP; T2: 10 g/kg of AOLP; T3: 15 g/kg of AOLP.

## Data Availability

The data presented in this study are available in the article.

## References

[1] Xu Q., Jian H., Zhao W., Li J., Zou X., Dong X. (2022). Early Weaning Stress Induces Intestinal Microbiota Disturbance, Mucosal Barrier Dysfunction and Inflammation Response Activation in Pigeon Squabs. Front. Microbiol..

[2] Yu S., Morris A., Kayal A., Milošević I., Van T., Sharma Bajagai Y., Stanley D. (2024). Pioneering Gut Health Improvements in Piglets with Phytogenic Feed Additives. Appl. Microbiol. Biotechnol..

[3] Moeser A.J., Pohl C.S., Rajput M. (2017). Weaning Stress and Gastrointestinal Barrier Development: Implications for Lifelong Gut Health in Pigs. Anim. Nutr..

[4] Rhouma M., Fairbrother J.M., Beaudry F., Letellier A. (2017). Post Weaning Diarrhea in Pigs: Risk Factors and Non-Colistin-Based Control Strategies. Acta Vet. Scand..

[5] Vinelli V., Biscotti P., Martini D., Del Bo’ C., Marino M., Meroño T., Nikoloudaki O., Calabrese F.M., Turroni S., Taverniti V. (2022). Effects of Dietary Fibers on Short-Chain Fatty Acids and Gut Microbiota Composition in Healthy Adults: A Systematic Review. Nutrients.

[6] Usuda H., Okamoto T., Wada K. (2021). Leaky Gut: Effect of Dietary Fiber and Fats on Microbiome and Intestinal Barrier. Int. J. Mol. Sci..

[7] Yin Y., Wang F., Yang M., Yin Y., Chen J., Yang Z. (2022). *Lycium barbarum* Polysaccharides as Antibiotic Substitutes Improve Growth Performance, Serum Immunity, Antioxidant Status, and Intestinal Health for Weaned Piglets. Front. Microbiol..

[8] Galgano S., Conway L., Fellows A., Houdijk J. (2024). Impact of Precursor-Derived Peracetic Acid on Post-Weaning Diarrhea, Intestinal Microbiota, and Predicted Microbial Functional Genes in Weaned Pigs. Front. Microbiol..

[9] Duarte M.E., Kim S.W. (2022). Phytobiotics from Oregano Extracts Enhance the Intestinal Health and Growth Performance of Pigs. Antioxidants.

[10] Zhu Q., Azad M.d.A.K., Li R., Li C., Liu Y., Yin Y., Kong X. (2024). Dietary Probiotic and Synbiotic Supplementation Starting from Maternal Gestation Improves Muscular Lipid Metabolism in Offspring Piglets by Reshaping Colonic Microbiota and Metabolites. mSystems.

[11] Omolaso B.O., Oluwole F.S., Odukanmi O.A., Adesanwo J.K., Ishola A.A., Adewole K.E. (2021). Evaluation of the Gastrointestinal Anti-Motility Effect of *Anacardium occidentale* Stem Bark Extract: A Mechanistic Study of Antidiarrheal Activity. J. Pharm. Anal..

[12] Oko A.O., Okose V.C., Ekuma E.T. (2022). *Anacardium occidentale* (Linn) Stem Bark Extracts: Effects on Poultry Colibacillosis Disease. Nig. J. Biotechnol..

[13] Hashim N.S., Tan M.L., Ooi K.L., Sulaiman S.F. (2023). The Effect of Flavonols in *Anacardium occidentale* L. Leaf Extracts on Skin Pathogenic Microorganisms. Nat. Prod. Res..

[14] Martínez Y., Martínez O., Liu G., Ren W., Rodríguez R., Fonseca Y., Olmo C., Isert M., Aroche R., Valdivié M. (2013). Effect of Dietary Supplementation with *Anacardium occidentale* on Growth Performance and Immune and Visceral Organ Weights in Replacement Laying Pullets. J. Food Agric. Environ..

[15] Aroche R., Martínez Y., Ayala L., Rodríguez R., Rodríguez Y. (2017). Growth Performance and Diarrhea Incidence in Post-Weaning Pigs, Supplemented with Plant Mixed Leaves Powder, with Nutraceutical Properties. Rev. Cien. Agri..

[16] Aroche R., Martínez Y., Ruan Z., Guan G., Waititu S., Nyachoti C.M., Más D., Lan S. (2018). Dietary Inclusion of a Mixed Powder of Medicinal Plant Leaves Enhances the Feed Efficiency and Immune Function in Broiler Chickens. J. Chem..

[17] Más D., Martínez Y., Rodríguez R., Salazar I., Aroche R., López B., Marcella D. (2016). Efecto de la Suplementación Dietética Con Polvos de Hojas de Guayaba (*Psidium guajava*) y Marañón (*Anacardium occidentale*) En El Comportamiento Productivo y la Incidencia de Diarrea En Cerdos Antes y Después del Destete. Rev. Comput. Prod. Porc..

[18] National Research Council (2012). Nutrient Requirements of Swine.

[19] Jiang X.R., Awati A., Agazzi A., Vitari F., Ferrari A., Bento H., Crestani M., Domeneghini C., Bontempo V. (2015). Effects of a Blend of Essential Oils and an Enzyme Combination on Nutrient Digestibility, Ileum Histology and Expression of Inflammatory Mediators in Weaned Piglets. Animal.

[20] Jiang X.R., Agazzi A., Awati A., Vitari F., Bento H., Ferrari A., Alborali G.L., Crestani M., Domeneghini C., Bontempo V. (2015). Influence of a Blend of Essential Oils and an Enzyme Combination on Growth Performance, Microbial Counts, Ileum Microscopic Anatomy and the Expression of Inflammatory Mediators in Weaned Piglets Following an *Escherichia coli* Infection. Anim. Feed Sci. Technol..

[21] Aroche R., Xian-Ren J., Bertot R., Li X., Avellaneda Barbosa M., Martínez Y. (2023). In Vitro Antimicrobial and Antioxidant Activity of Leaves and Aqueous Extract of Four Medicinal Plants with Phytobiotic Potential in Animal Production. Cuban J. Agric. Sci..

[22] Campbell J.M., Crenshaw J.D., Polo J. (2013). The Biological Stress of Early Weaned Piglets. J. Anim. Sci. Biotechnol..

[23] Sunderam V., Thiyagarajan D., Lawrence A.V., Mohammed S.S.S., Selvaraj A. (2019). In-Vitro Antimicrobial and Anticancer Properties of Green Synthesized Gold Nanoparticles Using *Anacardium occidentale* Leaves Extract. Saudi J. Biol. Sci..

[24] Duangjan C., Rangsinth P., Gu X., Wink M., Tencomnao T. (2019). Lifespan Extending and Oxidative Stress Resistance Properties of a Leaf Extracts from *Anacardium occidentale* L. in *Caenorhabditis elegans*. Oxid. Med. Cell. Longev..

[25] Siracusa R., Fusco R., Peritore A.F., Cordaro M., D’Amico R., Genovese T., Gugliandolo E., Crupi R., Smeriglio A., Mandalari G. (2020). The Antioxidant and Anti-Inflammatory Properties of *Anacardium occidentale* L. Cashew Nuts in a Mouse Model of Colitis. Nutrients.

[26] Chotphruethipong L., Benjakul S., Kijroongrojana K. (2017). Optimization of Extraction of Antioxidative Phenolic Compounds from Cashew (*Anacardium occidentale* L.) Leaves Using Response Surface Methodology. J. Food Biochem..

[27] Ukwenya V.O., Alese M.O., Ogunlade B., Folorunso I.M., Omotuyi O.I. (2023). *Anacardium occidentale* Leaves Extract and Riboceine Mitigate Hyperglycemia through Anti-Oxidative Effects and Modulation of Some Selected Genes Associated with Diabetes. J. Diabetes Metab. Disord..

[28] Shao Y., Peng Q., Wu Y., Peng C., Wang S., Zou L., Qi M., Peng C., Liu H., Li R. (2023). The Effect of an Essential Oil Blend on Growth Performance, Intestinal Health, and Microbiota in Early-Weaned Piglets. Nutrients.

[29] Ma L., Zhou B., Liu H., Chen S., Zhang J., Wang T., Wang C. (2024). Dietary Rutin Improves the Antidiarrheal Capacity of Weaned Piglets by Improving Intestinal Barrier Function, Antioxidant Capacity, and Cecal Microbiota Composition. J. Sci. Food Agric..

[30] May S., Knoell A., Vega C.G., Sardi M.I., Khafipour E. (2024). 192 Effects of Inclusion of a Probiotic and/or a Postbiotic in Diets Containing a Phytogenic Feed Additive on Growth Performance and Fecal Microbiome Composition and Function of Nursery Pigs. J. Anim. Sci..

[31] Liu S., Yunxia X., Cao S., Wen X., Xiao H., Li Y., Chi L., He D., Jiang Z., Wang L. (2022). Dietary Stevia Residue Extract Supplementation Improves Antioxidant Capacity and Intestinal Microbial Composition of Weaned Piglets. Antioxidants.

[32] Deng Z., Wang J., Wang J., Yan Y., Huang Y., Chen C., Sun L., Liu M. (2024). Tannic Acid Extracted from Gallnut Improves Intestinal Health with Regulation of Redox Homeostasis and Gut Microbiota of Weaned Piglets. Anim. Res. One Health.

[33] Zhu J., Lian J., Deng H., Luo J., Chen T., Sun J., Zhang Y.-L., Yang Y., Liu P., Xi Q. (2024). Effects of Spinach Extract and Licorice Extract on Growth Performance, Antioxidant Capacity, and Gut Microbiota in Weaned Piglets. Animals.

[34] Sun W., Chen Z., Huang Z., Wan A., Zhou M., Gao J. (2023). Effects of Dietary Traditional Chinese Medicine Residues on Growth Performance, Intestinal Health and Gut Microbiota Compositions in Weaned Piglets. Front. Cell. Infect. Microbiol..

[35] Zhuang Y., Wu H., Wang X., He J., He S., Yin Y. (2019). Resveratrol Attenuates Oxidative Stress-Induced Intestinal Barrier Injury through PI3K/Akt-Mediated Nrf2 Signaling Pathway. Oxid. Med. Cell. Longev..

[36] Zhou M., Ma J., Kang M., Tang W., Xia S., Yin J., Yin Y. (2023). Flavonoids, Gut Microbiota, and Host Lipid Metabolism. Eng. Life Sci..

[37] Li J., Zhang Q., Zhuo Y., Fang Z., Che L., Xu S., Feng B., Lin Y., Jiang X., Zhao X. (2022). Effects of Multi-Strain Probiotics and *Perilla frutescens* Seed Extract Supplementation Alone or Combined on Growth Performance, Antioxidant Indices, and Intestinal Health of Weaned Piglets. Animals.

[38] Xiao Y., Huang R., Wang N., Yuankun D., Yin Y., Qi M., Jing W. (2022). Ellagic Acid Alleviates Oxidative Stress by Mediating Nrf2 Signaling Pathways and Protects against Paraquat-Induced Intestinal Injury in Piglets. Antioxidants.

[39] Pierzynowska K., Woliński J., Weström B., Pierzynowski S.G. (2020). Maternal Immunoglobulins in Infants—Are They More than Just a Form of Passive Immunity?. Front. Immunol..

[40] Xiao C., Kai L., Teng C., Wei Z., Li J., Zhang S., Liu L., Lv H., Zhong R. (2023). Dietary Qi-Weng-Huangbo Powder Enhances Growth Performance, Diarrhoea and Immune Function of Weaned Piglets by Modulating Gut Health and Microbial Profiles. Front. Immunol..

[41] Abbas A.K., Lichtman A.H., Pillai S. (2021). Cellular and Molecular Immunology, 10e, South Asia Edition—E-Book.

[42] Zeng Y., Dai X., Chen Q., Liu Y., Bumbie G.Z., Sun W., Tang Z. (2022). Effect of Dietary Pomelo Peel Powder on Growth Performance, Diarrhea, Immune Function, Antioxidant Function, Ileum Morphology, and Colonic Microflora of Weaned Piglets. Animals.

[43] Fu L., Sun M., Dong W., Zhang G., Han D., Zang J., Liu H. (2022). Effects of Compound of Hawthorn (*Crataegus pinnatifida*) and Chinese Yam (*Dioscorea opposita* Thunb.) Extracts on Growth Performance, Intestinal Health, and Immune Function in Weaned Pigs. Anim. Sci. J..

[44] Mohammadi Gheisar M., Kim I.H. (2018). Phytobiotics in Poultry and Swine Nutrition—A Review. Ital. J. Anim. Sci..

[45] Johansson M.E.V., Hansson G.C. (2016). Immunological Aspects of Intestinal Mucus and Mucins. Nat. Rev. Immunol..

[46] Trachsel J., Briggs C., Gabler N.K., Allen H.K., Loving C.L. (2019). Dietary Resistant Potato Starch Alters Intestinal Microbial Communities and Their Metabolites, and Markers of Immune Regulation and Barrier Function in Swine. Front. Immunol..

[47] Suzuki T. (2020). Regulation of the Intestinal Barrier by Nutrients: The Role of Tight Junctions. Anim. Sci. J..

[48] Ma J., Rubin B.K., Voynow J.A. (2018). Mucins, Mucus, and Goblet Cells. CHEST.

[49] Wu Y., Zhang X., Liu X., Li Y., Han D., Pi Y., Whitmore M., Xingmiao L., Zhang G., Zheng J. (2023). Strain Specificity of Lactobacilli with Promoted Colonization by Galactooligosaccharides Administration in Protecting Intestinal Barriers during *Salmonella* Infection. J. Adv. Res..

[50] Sartor R.B., Mazmanian S.K. (2012). Intestinal Microbes in Inflammatory Bowel Diseases. Am. J. Gastroenterol. Suppl..

[51] Lozupone C.A., Stombaugh J.I., Gordon J.I., Jansson J.K., Knight R. (2012). Diversity, Stability and Resilience of the Human Gut Microbiota. Nature.

[52] Heo J.M., Opapeju F.O., Pluske J.R., Kim J.C., Hampson D.J., Nyachoti C.M. (2013). Gastrointestinal Health and Function in Weaned Pigs: A Review of Feeding Strategies to Control Post-Weaning Diarrhoea without Using in-Feed Antimicrobial Compounds. J. Anim. Physiol. Anim. Nutr..

[53] Priyadarshini M., Kotlo K.U., Dudeja P.K., Layden B.T. (2018). Role of Short Chain Fatty Acid Receptors in Intestinal Physiology and Pathophysiology. Compr. Physiol..

